# Dissection Around the Superior Mesenteric Artery (SMA) Using LigaSure Maryland During Left Posterior Approach for Pancreaticoduodenectomy

**DOI:** 10.7759/cureus.27034

**Published:** 2022-07-19

**Authors:** Masatoshi Kajiwara, Ryo Nakashima, Takahide Sasaki, Shigetoshi Naito, Suguru Hasegawa

**Affiliations:** 1 Gastroenterological Surgery, Faculty of Medicine, Fukuoka University, Fukuoka, JPN

**Keywords:** ligasure, mesopancreas, left posterior approach, pancreaticoduodenectomy, pancreatic head cancer

## Abstract

R0 resection for pancreatic head cancer without exposing the tumor demands complete resection of “mesopancreas”. In other words, dividing the proximal jejunal artery and vein at their roots of the superior mesenteric artery (SMA) and superior mesenteric vein (SMV) respectively during pancreaticoduodenectomy (PD) is absolutely essential. We present here our standardized dissection procedures around the SMA during the left posterior approach for PD. This procedure is safe and reproducible owing to the secure sealing performance of LigaSure^TM^ Maryland.

## Introduction

The mortality rate of pancreatic cancer is still high, regardless of recent advances in multidisciplinary treatment including surgical resection, chemotherapy, and radiotherapy [1,2]. Surgery remains an important part of these treatments and curative R0 resection for pancreatic cancer has been reported to be an independent prognostic factor for improved survival [3,4]. R0 resection for pancreatic head cancer demands complete resection of “mesopancreas” [5,6], which implies dividing the proximal jejunal artery (JA) and jejunal vein (JV) at their roots of the superior mesenteric artery (SMA) and superior mesenteric vein (SMV) respectively during pancreaticoduodenectomy (PD). PD is considered one of the most complicated abdominal surgeries with high mortality and morbidity rates [7].

The left posterior approach is one of the “artery-first” approaches for PD and is especially suitable for uncinate process pancreatic cancer (UPPC) [8,9]. It allows extensive exposure of the SMA for a length of approximately 6-8 cm from its origin [9]. However, the detailed procedures of the left posterior approach for PD have not been well-documented so far. We herein present our standardized vessel-guided dissection procedures around the SMA during the left posterior approach for PD using LigaSure^TM^ Maryland (Medtronics, Minneapolis, MN) with video.

## Technical report

A 64-years-old female patient underwent PD for the UPPC. Preoperative images revealed that the tumor was 19 mm in size without vascular or lymph node involvement. 

**Figure 1:**
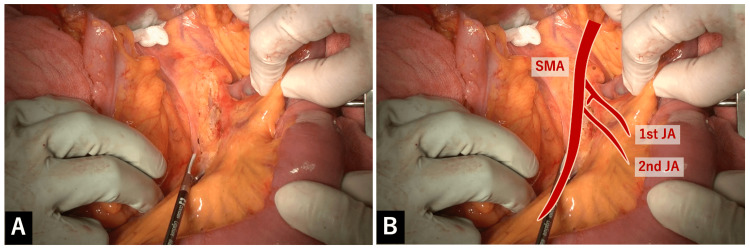
Separation of serosal membrane of the mesentery of the proximal jejunum above the SMA A: In the elevated view of the transverse colon, serosal membrane of the mesentery of the proximal jejunum was separated directly above the SMA. The underlying fat layer was then carefully dissected layer by layer with LigaSure^TM^ Maryland. B: Overlay image for the explanation of A.

In the elevated view of the transverse colon, serosal membrane of the mesentery of the proximal jejunum was separated directly above the SMA. The underlying fat layer was then carefully dissected layer by layer with LigaSure^TM^ Maryland (23cm for open surgery) (Figure 1).

**Figure 2:**
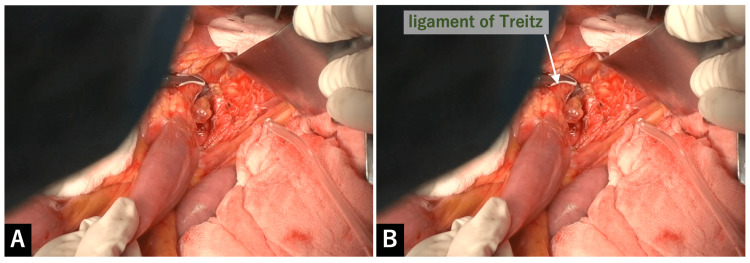
Transection of the ligament of Treitz A: The soft tissue on the cranial side of the duodenojejunal flexure was dissected, then, the ligament of Treitz was identified on its dorsal side. The ligament of Treitz was transected as cranially as possible. B: Explanation figure of A.

Appropriate traction applied to the mesentery with the surgeon's left hand and the assistant's hand facilitates the dissection. The soft tissue on the cranial side of the duodenojejunal flexure was dissected, then, the ligament of Treitz was identified on its dorsal side. The ligament of Treitz was transected as cranially as possible (Figure 2).

**Figure 3:**
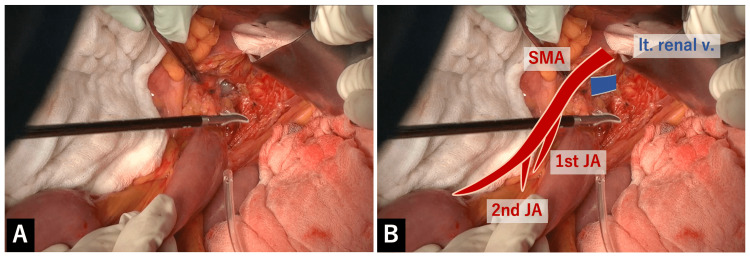
The left-to-dorsal wall of the root of the SMA and the left renal vein A: Behind the ligament of Treitz was the left-to-dorsal wall of the root of the SMA covered by nerve plexus and the left renal vein. This will be the cranial landmark of the dissection around the SMA. B: Overlay image for the explanation of A.

Behind the ligament of Treitz was the left-to-dorsal wall of the root of the SMA covered by nerve plexus and the left renal vein. This will be the cranial landmark of the dissection around the SMA (Figure 3).

**Figure 4:**
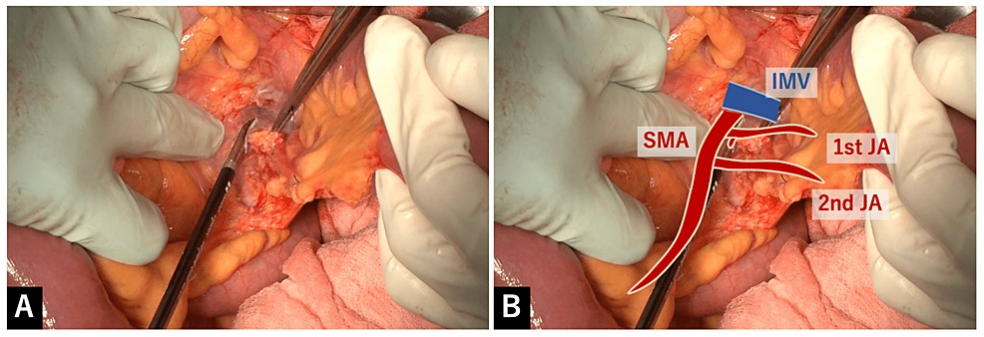
Dissection of the fatty tissue on the left-to-dorsal side of the SMA A: After the jejunum was transected, the fatty tissue on the left-to-dorsal side of the SMA was dissected. While checking the running course of the SMA as a guide, the left-to-dorsal side of the SMA covered by nerve plexus was exposed toward the point where the ligament of Treitz was taken down. B: Overlay image for the explanation of A.

After the jejunum was transected, the fatty tissue on the left-to-dorsal side of the SMA was dissected. While checking the running course of the SMA as a guide, the left-to-dorsal side of the SMA covered by nerve plexus was exposed toward the point where the ligament of Treitz were taken down (Figure 4).

**Figure 5:**
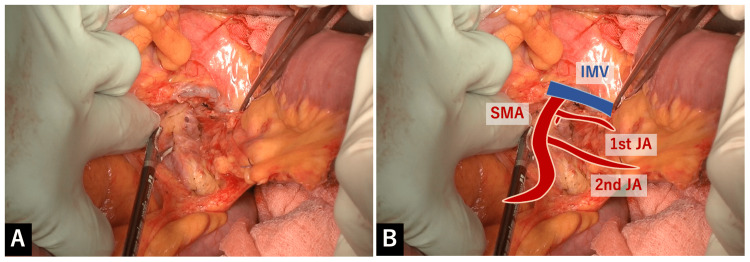
Clarifying how the proximal (1st and 2nd) JAs run inside the mesentery A: Although many minute blood vessels can be seen in the mesentery of the jejunum, these vessels were securely sealed with LigaSure^TM^ Maryland. Gradually, it became clear how the proximal (1st and 2nd) JAs run inside the mesentery. B: Overlay image for the explanation of A.

Although many minute blood vessels can be seen in the mesentery of the jejunum, these vessels were securely sealed with LigaSure^TM^ Maryland. Gradually, it became clear how the proximal (1st and 2nd) JAs run inside the mesentery (Figure 5).

**Figure 6:**
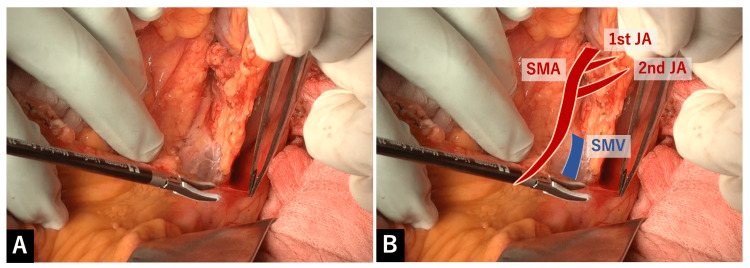
Exposure of the posterior wall of the SMV at the dorsal side of the jejunal mesentery A: We tried to find the posterior wall of the SMV at the dorsal side of the jejunal mesentery and expose it cranially. B: Overlay image for the explanation of A.

Next, we tried to find the posterior wall of the SMV at the dorsal side of the jejunal mesentery and expose it cranially (Figure 6). 

**Figure 7:**
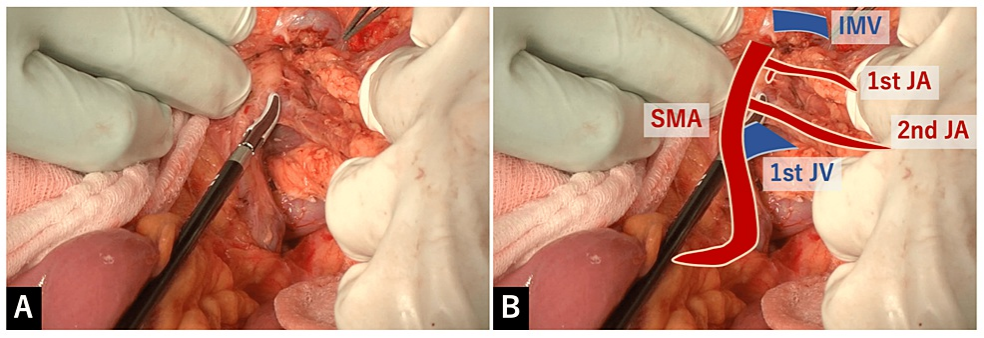
Transection of the nerves around the root of the JAs on the ventral side of the mesentery A: Returning to the ventral side of the mesentery, the nerves near the JAs' roots were severed in order to divide them. B: Overlay image for the explanation of A.

Returning to the ventral side of the mesentery, the nerves near the JAs' roots were severed in order to divide them (Figure 7).

**Figure 8:**
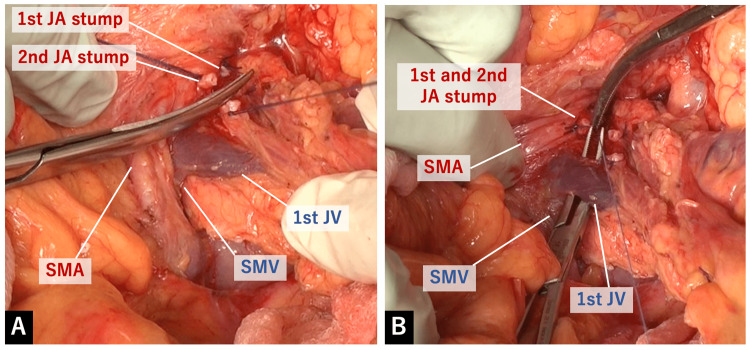
Division of the proximal JAs and JV A: The 1st and 2nd JAs were ligated and separated at their roots of the SMA. B: Behind it was the junction of the proximal (1st) JV and the SMV without distortion, and the 1st JV was safely ligated and divided.

The 1st and 2nd JAs were ligated and separated at their roots of the SMA. Behind it was the junction of the proximal (1st) JV and the SMV without distortion, and the 1st JV was safely ligated and divided (Figure 8).

The left posterior approach for PD lasted 32min with a small amount of bleeding (Video 1).

**Video 1:** SMA dissection using LigaSure Maryland during the left posterior approach of pancreaticoduodenectomy

Pathological examination revealed R0 resection of the tumor (adenocarcinoma, T2) and no lymph node involvement. The postoperative course was uneventful without readmission and there is no sign of recurrence during 18-month follow-up.

## Discussion

**Figure 9:**
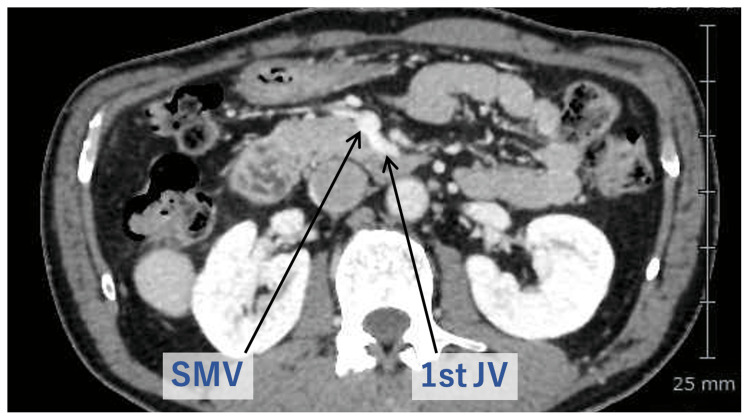
Relationship between the 1st JV and the SMV The proximal (1st) JV usually drains into the SMV at its left posterior side. JV: Jejunal vein; SMV: Superior mesenteric vein

It is not always easy to accomplish R0 resection of pancreatic head cancer, which is mandatory for prolonged survival. One of the reasons is the complex anatomy around the SMA and SMV due to “intestinal rotation”. Moreover, the injury of the proximal JV will result in massive bleeding which makes subsequent accurate surgery difficult. The proximal (1st) JV usually drains into the SMV at its left posterior side (Figure 9).

Among several “artery-first” approaches for PD, the left posterior approach enables identifying the junction of the proximal JV and the SMV without distortion (Figure 8). As the proximal JV is reported to be an anatomical landmark for the caudal border of mesopancreas [10], it is important to focus on safely identifying the root of the SMV. In most cases (more than 70% [11]), the proximal JV passes behind the SMA. Therefore, it is necessary to divide the proximal JA to reach the root of the proximal JV. In this respect as well, the left posterior approach has an advantage because the SMA is widely exposed and the root of the proximal JA can be securely divided in a good surgical view. Being aware of the running direction of the SMA guides and helps us know how the proximal JA runs inside the mesentery and find its root to be divided.

LigaSure^TM^, an advanced bipolar vessel sealing system, has been widely used in the field of abdominal surgery, including PD [12,13]. The high sealing effect of LigaSure^TM^ enables reliable hemostasis for minutes vessels within the mesentery of the jejunum, and its low collateral thermal effect [14] is suitable for application against adjacent vessels. Moreover, the smooth-tapered tip of LigaSure^TM^ Maryland is optimal for carefully dissecting fat tissues and nerves in the mesentery layer by layer despite the complex vascular anatomy around the SMA. 

The combination of the left posterior approach and LigaSure^TM^ Maryland requires no ligation except for dividing the proximal JA and JV during the left posterior approach. This means our procedure might be applicable to minimally invasive (laparoscopic or robotic) surgery.

## Conclusions

The left posterior approach for PD using LigaSure^TM^ Maryland is safe and feasible. It helps efficiently accomplish R0 resection of pancreatic head cancer. High sealing performance of LigaSure^TM^ Maryland can reduce the chance of clipping or tying, which means this procedure can also be applied to minimally invasive (laparoscopic or robotic) surgery.
